# Transcriptional profiling reveals multiple defense responses in downy mildew-resistant transgenic grapevine expressing a TIR-NBS-LRR gene located at the *MrRUN1*/*MrRPV1* locus

**DOI:** 10.1038/s41438-021-00597-w

**Published:** 2021-07-01

**Authors:** Junjie Qu, Ian Dry, Lulu Liu, Zexi Guo, Ling Yin

**Affiliations:** 1 Guangxi Crop Genetic Improvement and Biotechnology Key Lab, Guangxi Academy of Agricultural Sciences, Nanning, 530007 China; 2 CSIRO Agriculture & Food, Wine Innovation West Building, Locked Bag 2, Glen Osmond, SA 5064 Australia

**Keywords:** Biotic, Microbe

## Abstract

Grapevine downy mildew (DM) is a destructive oomycete disease of viticulture worldwide. *MrRPV1* is a typical TIR-NBS-LRR type DM disease resistance gene cloned from the wild North American grapevine species *Muscadinia rotundifolia*. However, the molecular basis of resistance mediated by *MrRPV1* remains poorly understood. Downy mildew-susceptible *Vitis vinifera* cv. Shiraz was transformed with a genomic fragment containing *MrRPV1* to produce DM-resistant transgenic Shiraz lines. Comparative transcriptome analysis was used to compare the transcriptome profiles of the resistant and susceptible genotypes after DM infection. Transcriptome modulation during the response to *P. viticola* infection was more rapid, and more genes were induced in *MrRPV1*-transgenic Shiraz than in wild-type plants. In DM-infected *MrRPV1*-transgenic plants, activation of genes associated with Ca^2+^ release and ROS production was the earliest transcriptional response. Functional analysis of differentially expressed genes revealed that key genes related to multiple phytohormone signaling pathways and secondary metabolism were highly induced during infection. Coexpression network and motif enrichment analysis showed that WRKY and MYB transcription factors strongly coexpress with stilbene synthase (*VvSTS*) genes during defense against *P. viticola* in *MrRPV1*-transgenic plants. Taken together, these findings indicate that multiple pathways play important roles in *MrRPV1-*mediated resistance to downy mildew.

## Introduction

Plants have evolved complex and sophisticated defense mechanisms to cope with the threat of pests and diseases. These defense mechanisms can be divided into two layers of immune responses, PAMP-triggered immunity (PTI) and effector-triggered immunity (ETI)^1^. PTI is the first layer of immune defense and is triggered the detection of conserved microbe- or pathogen-associated molecular patterns (MAMPs/PAMPs) or self-molecules (damage-associated molecular patterns, DAMPs) by pattern-recognition receptors (PRRs)^2^. ETI is activated by pathogen effector proteins via intracellular immune receptors, which typically possess central nucleotide binding and C-terminal leucine-rich repeat domains (NLRs)^3^. The intracellular immune receptors involved in ETI are also known as plant disease resistance (R) proteins. Immune receptors can be subdivided into two classes based on whether their N-terminal domain shares homology with cytosolic domains of Drosophila Toll or animal interleukin-1 receptors (TIR-NBS-LRR) or has a predicted coiled-coil domain (CC-NBS-LRR)^4^.

Grapevine downy mildew (DM), caused by the oomycete *Plasmopara viticola*^5,6^, is a serious disease of viticulture worldwide. The resistance of grapes to DM varies between Vitaceae genera and species^7^. To date, a total of 31 quantitative trait loci (QTLs) from different *Vitis* and *Muscadinia* genotypes that confer resistance to DM have been genetically mapped to chromosomes 4, 5, 7, 9, 12, 14, 15, and 18^8–17^. However, only two resistance genes associated with these QTLs that mediate the resistance response have been identified. DM resistance conferred by the *MrRUN1/MrRPV1* locus from *Muscadinia rotundifolia* is mediated by a single TIR-NBS-LRR (TNL)-type resistance gene designated *MrRPV1*^18^. The *Rpv3-1* locus, on the other hand, which is derived from a North American *Vitis* species, may require the involvement of two tandemly duplicated TNL-type genes^19^.

Studies examining the immune response to DM infection have been described in a number of DM-resistant grapevine species^20–26^. Most of these studies have focused on comparing the differences in the infection process between susceptible and resistant varieties and include morphological and histological differences, as well as differences in transcriptional responses. For example, leaf hairs of some grape varieties can form a natural physical barrier to prevent the invasion of the DM pathogen^27^. Transcriptional analysis of *V. riparia*^20^, *V. amurensis*^21,22,24^, *V. pseudoreticulata*^26^, *M. rotundifolia*^28^, *V. labrusca*, and *V. vinifera* hybrids containing DM resistance loci^23,24,29^ has revealed that the induction and regulation of many defense-related genes may contribute to DM resistance. However, one observation that is common across many of these studies is that there is more rapid or stronger upregulation of stilbene biosynthesis pathway genes in DM-resistant genotypes than in DM-susceptible genotypes. For example, Wang et al.^28^ showed that stilbene synthase (*VvSTS*) was induced earlier in DM-resistant *M. rotundifolia* than in susceptible *V. vinifera* tissues following DM inoculation, while the resveratrol *O*-methyltransferase (*ROMT*) gene was upregulated in only the resistant genotype. More recent studies with *V. vinifera* hybrids containing either the *Rpv3* or *Rpv10* resistance locus have also strongly suggested a role for stilbenes in DM resistance mediated by these loci^23,24,29^. Similarly, in *Rpv12*-mediated DM resistance, trans-resveratrol was proposed to act as a signaling molecule in ROS formation and initiation of programmed cell death (PCD) triggered by a CC-NB-LRR gene product^24^.

One of the drawbacks of many of these studies is that they involve comparisons of DM-induced gene expression profiles between resistant and susceptible individuals, which not only differ in the presence/absence of a DM resistance locus but also have different genetic backgrounds, i.e., they compare either different individuals from a segregating population^22,29^, different *Vitis* cultivars^23^ or completely different grapevine species^20,25,28^. This makes it difficult to conclude with certainty that all of the differences in DM-induced gene expression observed between the resistant and susceptible genotypes are mediated by the DM resistance locus alone or are a function of other genotypic differences.

Feechan et al.^18^ previously reported on the map-based cloning of seven putative resistance genes (RGAs) located at an *MrRUN1*/*MrRPV1* locus introgressed from the wild North American grape species *M. rotundifolia*. Functional testing of these *MrRGA* genes for mildew resistance revealed that *MrRGA8* conferred resistance to *P. viticola*, and *MrRGA8* was designated *MrRPV1*, while *MrRGA10* conferred resistance to *Erysiphe necator* and was designated *MrRUN1*. In this study, we used a transgenic DM-resistant Shiraz line expressing the *MrRPV1* gene (S8-1) and wild-type plants of the same Shiraz clone, which lack the *MrRPV1* gene, to investigate *MrRPV1*-mediated transcriptome responses within the first 36 h after DM inoculation. A weighted gene coexpression network analysis (WGCNA) was performed to identify the hub genes and key pathways involved in *MrRPV1*-mediated resistance. This information has allowed us to draw conclusions regarding the molecular mechanisms underlying DM resistance conferred strictly by the *MrRPV1* gene without the complications associated with previous studies based on comparisons of transcriptional responses in plants with different genetic backgrounds.

## Results

### *MrRPV1*-mediated transcriptional responses to DM infection

Feechan et al.^18^ previously showed that transgenic versions of the *V. vinifera* cultivars Portan, Tempranillo, and Shiraz expressing the *MrRPV1* gene displayed a 92–98% reduction in DM sporulation at 6 days postinoculation compared to the wild-type controls. The strongest resistance was observed in Shiraz transgenic line S8-1, which was associated with the induction of PCD in penetrated cells. The transcriptional responses of leaf discs of *MrRPV1*-transgenic Shiraz line S8-1 to *P. viticola* inoculation were thus compared with those of wild-type Shiraz leaf discs lacking the *MrRPV1* gene. An examination of the timing of *MrRPV1*-mediated PCD induction in transgenic Shiraz, as determined by staining with trypan blue^18^, indicated that it was first observed 24 h post inoculation (hpi) (data not shown), and the transcriptional response within the first 36 hpi was therefore made a focus. Both the Shiraz transgenic line S8-1 and a wild-type Shiraz plant were propagated to produce six clonal copies of each, which were divided into three replicates of two plants each from which leaves were sampled to obtain leaf discs. Leaf discs were inoculated with DM sporangia or water and sampled at 0, 12, 18, 24, and 36 h. The remaining discs were incubated for an additional 4–5 days to check for sporulation; no sporulation was observed on the S8-1 leaf discs, whereas leaf discs from the wild-type Shiraz showed a dense covering of sporangia (~1.3 × 10^5^ sporangia per disc).

**Fig. 1:**
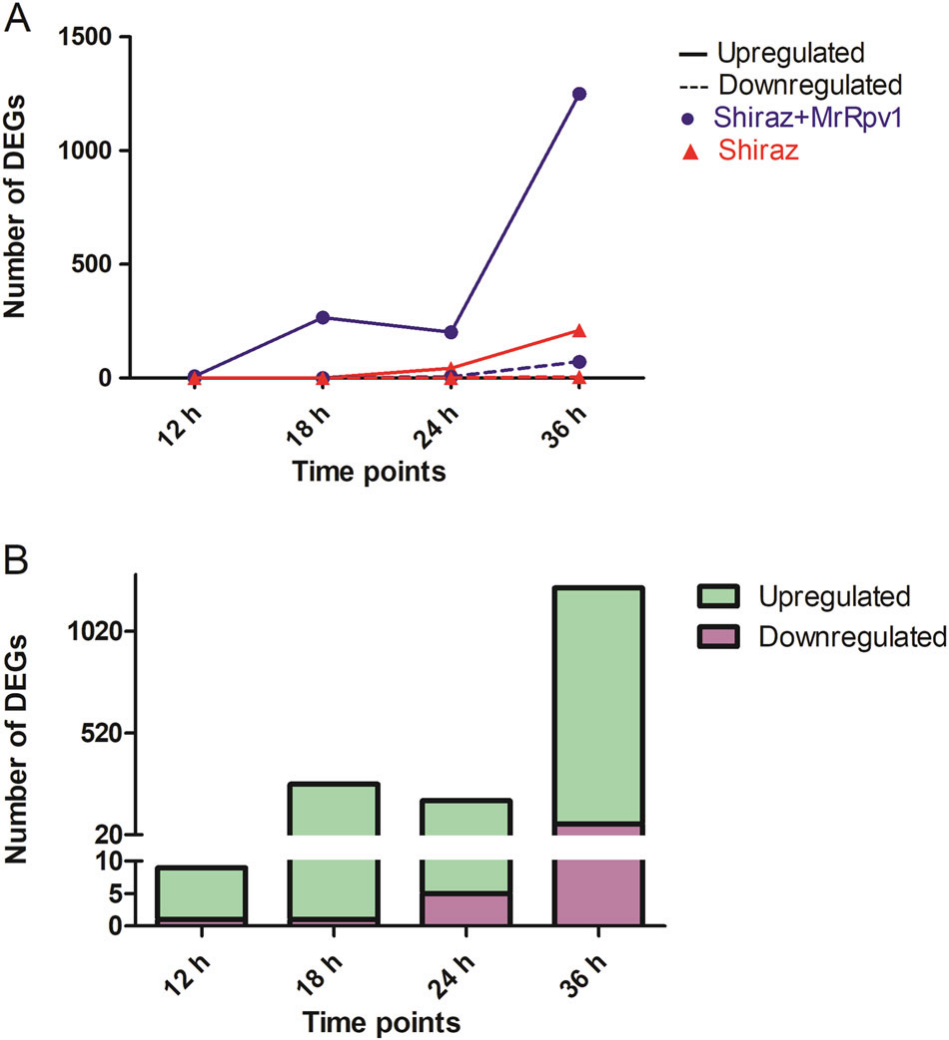
Transcriptome dynamics in wild-type Shiraz or *MrRPV1*-transgenic Shiraz challenged with *Plasmopara viticola*. **A** The number of DEGs was plotted at each time point in wild-type Shiraz and *MrRPV1*-transgenic Shiraz challenged with *P. viticola* compared with mock-inoculated samples. **B** The number of up- and downregulated genes in *MrRPV1*-transgenic Shiraz compared with wild-type Shiraz after *P. viticola* infection

For the convenience of description, S_Mock and TS_Mock are used to represent the wild-type Shiraz plants and *MrRPV1*-transgenic Shiraz plants under control conditions, whereas S_DM and TS_DM represent DM-infected plants, respectively. To compare transcription profiles between wild-type Shiraz plants and *MrRPV1*-transgenic Shiraz plants in response to DM inoculation, we subjected the expression data to pairwise comparisons, i.e., S_DM vs. S_Mock and TS_DM vs. TS_Mock, at different time points (Supplemental Data Set 1). Figure 1A shows that the number of differentially expressed genes (DEGs) that were either upregulated or downregulated in the comparison of TS_DM vs. TS_Mock increased from 9 at 12 hpi to 1322 at 36 hpi. In contrast, in the comparison of S_DM vs. S_Mock, no DEGSs were observed at 12 hpi or 18 hpi, but the number increased to 45 at 24 hpi and 216 at 36 hpi. In addition, 180 DEGs were shared between S_DM vs. S_Mock and TS_DM vs. TS_Mock (Fig. S1). Furthermore, 85 out of these 180 common genes were present at 12 hpi and 18 hpi in *MrRPV1*-transgenic Shiraz plants (Supplemental Data Set 2). This finding indicates that DM effector recognition by MrRPV1, either directly or indirectly through interaction with an effector-modified guard or decoy protein^30^, results in a more rapid and extensive transcriptomic response to *P. viticola* infection in Shiraz cells with MrRPV1 than in Shiraz cells lacking MrRPV1.

To gain a better understanding of the mechanism of resistance mediated specifically by the *MrRPV1* gene, we focused on a subset of DEGs that had to meet one of the following criteria: (1) the genes were differentially expressed in the comparison of TS_DM vs. TS_Mock but not differentially expressed in the comparison of S_DM vs. S_Mock or (2) the genes are differentially expressed in both S_DM vs. S_Mock and TS_DM vs. TS_Mock, but the differences in TS_DM vs. TS_Mock group were statistically significantly different from the S_DM vs. S_Mock comparison values (*P*-value of Fisher’s test <0.05). This refinement resulted in the identification of a total of 1356 DEGs at four-time points (Supplemental Data Set 3). Almost all of the DEGs were differentially expressed in transgenic plants specifically in response to DM infection at at least one-time point. Only seven DEGs met the second criterion, i.e., were significantly induced in response to DM in both *MrRPV1*-transgenic and wild-type Shiraz leaf discs but were significantly more highly induced in the presence of *MrRPV1*. Furthermore, 94% of the DEGs were upregulated, with only 6% significantly downregulated (Fig. 1B). These findings indicate that the following activation in the presence of DM*, MrRPV1* functions mainly as a positive regulator of gene expression to mediate DM resistance. Quantitative real-time PCR (qPCR) assays of a set of randomly selected DEGs confirmed that their expression was in accordance with the results of the transcriptome analysis (Fig. S2).

**Fig. 2:**
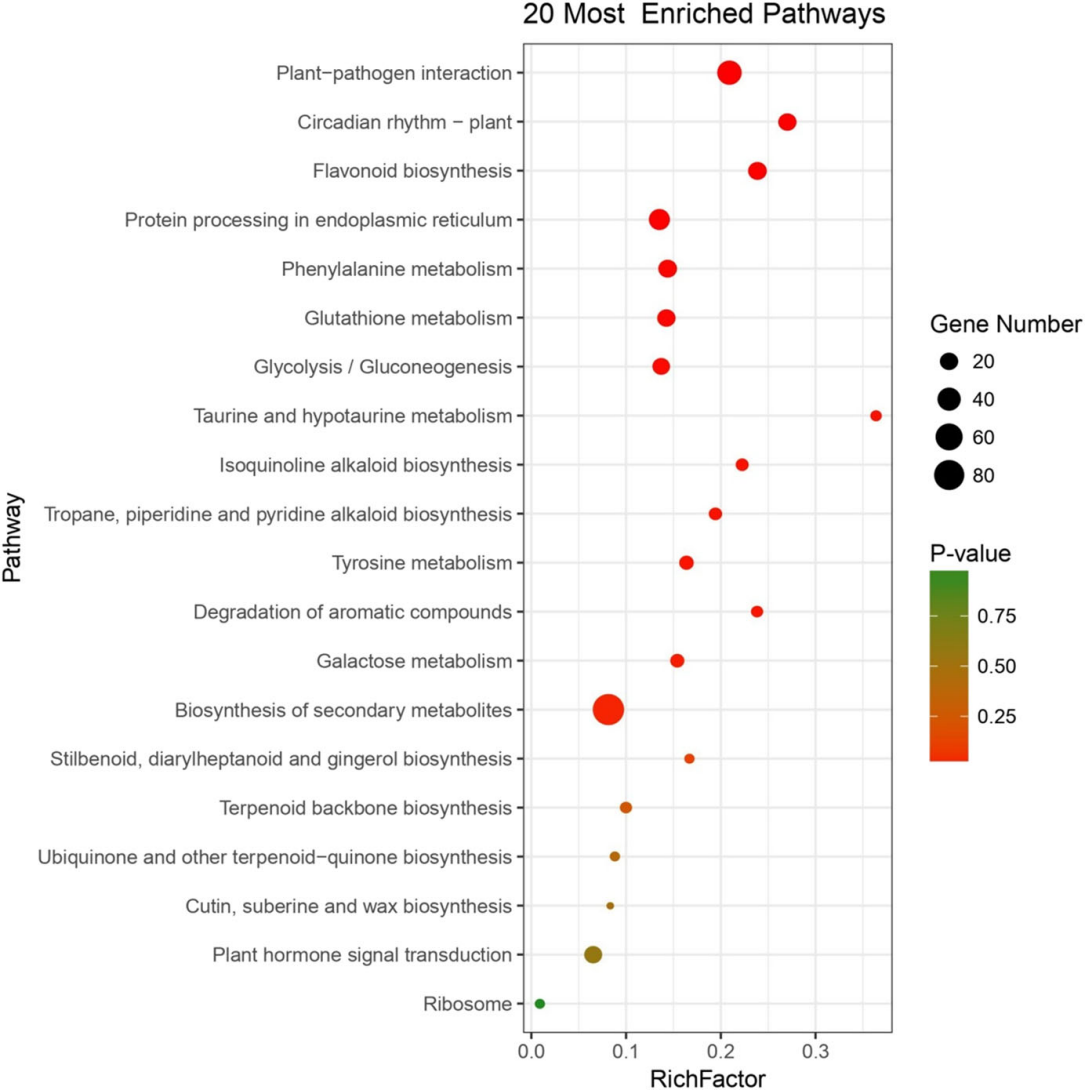
The top 20 significantly enriched KEGG pathways of DEGs in *MrRPV1-*transgenic lines. RichFactor refers to the ratio of the number of genes located in the pathway entry in the DEGs to the total number of annotated genes located in the pathway entry. The greater RichFactor is, the greater the degree of enrichment. The *q*-value indicates the *P*-value after correction for the testing of multiple hypotheses. The range of the *q*-value is [0, 1], with values closer to zero indicating more significant enrichment

Gene ontology (GO) and Kyoto Encyclopedia of Genes and Genomes (KEGG) pathway enrichment analyses of these DEGs were performed based on the timing of expression changes. No significant GO enrichment terms were obtained at 12 hpi because there were too few DEGs. The GO enrichment analysis of DEGs at later time points (18–36 hpi) revealed that genes involved in biological processes related to metabolic processes, including protein phosphorylation and protein modification, were significantly enriched (Table S4). Among the molecular function terms, most were related to plant immunity, including protein kinase activity, phosphotransferase activity, ATP or nucleoside binding, calcium ion binding, lipase activity, etc. (Supplemental Data Set 4). KEGG results showed that most of the DEGs in *MrRPV1*-transgenic lines were enriched in biological pathways related to biotic stress (Fig. 2), including biosynthesis of secondary metabolites, plant-pathogen interaction, protein processing in the endoplasmic reticulum, flavonoid biosynthesis, phenylalanine metabolism, glutathione metabolism, and glycolysis/gluconeogenesis.

### DEGs related to reactive oxygen species

Reactive oxygen species (ROS) are widely produced in many plants with a transient burst as an early and rapid response to pathogen attack. Our results showed differential expression of several categories of respiratory burst genes involved in ROS production and balance, including genes encoding peroxidases, oxidases, and glutathione S-transferases (Supplemental Data Set 5). Within 12 h of DM inoculation in *MrRPV1*-transgenic plants, two genes encoding nonsymbiotic hemoglobin and peroxidase showed ~2.5-fold and ~2.9-fold increases compared with their expression levels in the mock control. At later time points (18–36 hpi), significant upregulation of the genes encoding six L-ascorbate oxidases (~2.7- to ~103.9-fold), two respiratory burst oxidase homolog (RBOH) proteins (~2.1-fold and ~8.2-fold) and 20 glutathione S-transferases (GSTs, ~1.8- to ~138.0-fold) was observed in *MrRPV1*-transgenic leaf discs. For example, the expression of an *L-ascorbate oxidase* (VIT_07s0031g01070) showed an ~12.9-fold increase at 18 hpi, peaked at an increase of ~103.9-fold at 24 hpi and decreased ~45-fold at 36 hpi compared to expression in mock control leaf discs.

### DEGs related to calcium- and kinase-mediated signaling

Kinase-mediated signaling plays a vital role in plant innate immunity. In our study, more than two hundred protein kinases, including *receptor-like protein kinases* (*RLKs*), *mitogen-activated protein kinases* (*MAPKs* or *MPKs*), and *wall-associated receptor kinases* (*WAKs*), were more highly upregulated in the *MrRPV1*-transgenic lines than in the wild-type control lines within 36 h after inoculation with DM (Supplemental Data Set 6). These differentially expressed *RLKs* included three types: *cysteine-rich receptor-like protein kinases* (*CRKs*), *L-type lectin-domain containing receptor kinases* (*LecRKs*) and *G-type lectin S-receptor-like serine/threonine-protein kinases*. Among them, there were 47 *RLKs* with greater than a 10-fold change in expression. The two most highly upregulated *RLK* genes VIT_09s0002g03010 and VIT_19s0014g04190 were both with a ~108.9-fold increase at 36 hpi in *MrRPV1*-transgenic plants after *P. viticola* infection. Recognition of PAMPs by their receptors triggers a series of downstream defense responses, including the activation of MAPKs or MPKs, calcium influx, and upregulation of defense genes. From 24 hpi, differential expression of MAPK was detected with slight changes (~1.6- to ~3.8-fold). Significant upregulation of the genes encoding four types of calcium sensors, including *calcium cyclic nucleotide-gated ion channel* (*CNGC*), *calcium/calmodulin-binding protein* (*CBP*), *calcium-dependent protein kinase* (*CDPK*) and *CBL-interacting protein kinase* (*CIPK*), was observed in only the *MrRPV1*-transgenic line. In addition, one calcium-transporting ATPase (Novel00720) and three calcium-binding proteins, CML (VIT_01s0010g02930, VIT_01s0010g02940, VIT_01s0010g02970), were expressed only after DM infection in the *MrRPV1*-transgenic line. During pathogen infection, WAKs trigger the innate immune response as candidate receptors of cell wall-associated oligogalacturonides^31^. The expression levels of two *WAKs* (VIT_03s0132g00340 and VIT_03s0132g00350) continuously increased compared with those of the control from 18 to 36 hpi. Another *WAK* gene, VIT_17s0000g04420, was expressed only after DM infection in the *MrRPV1*-transgenic line.

### *RPV1*-mediated activation of key genes related to hormone biosynthesis and signaling pathways

The plant hormones salicylic acid (SA)^32^, jasmonate (JA)^33^, ethylene (ET)^34^, and auxin^35^ play important roles in plant resistance to oomycete pathogens. In our study, 1-aminocyclopropane-1-carboxylate synthase (ACS1) and 1-aminocyclopropane-1-carboxylate oxidase (ACO), the two key enzymes in ethylene biosynthesis, showed elevated expression levels in response to DM inoculation (Supplemental Data Set 7). One *ACS1* gene (VIT_15s0046g02220) was induced in only *MrRPV1*-transgenic plants at 36 hpi. Two *ACO* genes (VIT_05s0049g00430, VIT_12s0059g01380) were upregulated significantly at 18 hpi (~3.0-fold), and three *ACO* genes (VIT_05s0049g00430, VIT_12s0059g01380 and VIT_05s0049g00410) were upregulated significantly at 36 hpi (~2.7- to ~7.1-fold) in *MrRPV1*-transgenic plants. A set of genes encoding ethylene-responsive transcription factors (Supplemental Data Set 10) was also significantly upregulated from 18 h to 36 h after infection in *MrRPV1*-transgenic plants. For jasmonate biosynthesis, upregulation of genes encoding an allene oxide synthase (AOS) (~4.5-fold) and three lipoxygenases (LOXs) (~3.0- to ~9.6-fold) was identified at 36 hpi.

The genes encoding EDS, PAD4, and SAG101, which are important for signal transduction mediated by TNL-R-protein, were upregulated at 18 hpi (~2.4-fold) and 36 hpi (~1.6- to ~6.7-fold) (Supplemental Data Set 7). The increased expression of ten PR genes, including *PR-1*, *PR-4,* and *STH-21*, was also observed in *MrRPV1*-transgenic plants in response to *P. viticola* attack. It is particularly noteworthy that the expression of *PR1* (VIT_03s0088g00890) was increased ~478.0-fold at 36 hpi compared with the mock-inoculated transgenic plants (Table S3). The expression of another *PR1* gene (VIT_03s0088g00700) was observed only after DM infection in *MrRPV1*-transgenic lines. Three *SAR DEFICIENT 1* (*SARD1*) genes, which have been reported as key regulators for *ICS1* induction and SA synthesis^36^, were upregulated ~2.8-, ~6.3-, and ~12.4-fold at 18 hpi. Auxin is generally a negative regulator in plant disease resistance^37^. Consistent with this, the expression of the auxin-responsive gene *SAUR22* was downregulated in *MrRPV1*-transgenic lines in response to DM inoculation.

### *RPV1*-mediated activation of genes related to secondary metabolism

Many studies have shown that secondary metabolites, including phenolic compounds, terpenes, and alkaloids, may play a role in plant defense against pathogens^38^. In the current research, the pathways related to secondary metabolite biosynthesis, including flavonoid biosynthesis; tropane, piperidine, and pyridine alkaloid biosynthesis; isoquinoline alkaloid biosynthesis; stilbenoid, diarylheptanoid, and gingerol biosynthesis; ubiquinone and other terpenoid-quinone biosynthesis; and terpenoid backbone biosynthesis pathways, are represented (Fig. 2). A total of 18 stilbene synthases (*STSs*) were upregulated within 36 hpi in *MrRPV1*-transgenic leaves, with 17 of them showing increases ranging from ~3.0- to ~24.9-fold at 18 hpi (Supplemental Data Set 8). Seven phenylalanine ammonia-lyases (*PALs*) were also upregulated by 2.9-fold to ~16.3-fold, and two *ROMT* genes were upregulated by 30.1- and 93.3-fold in *MrRPV1*-transgenic leaves at 36 hpi by DM compared to mock-inoculated leaves (Supplemental Data Set 8).

### Coexpression network analysis to identify key modules and hub genes

**Fig. 3:**
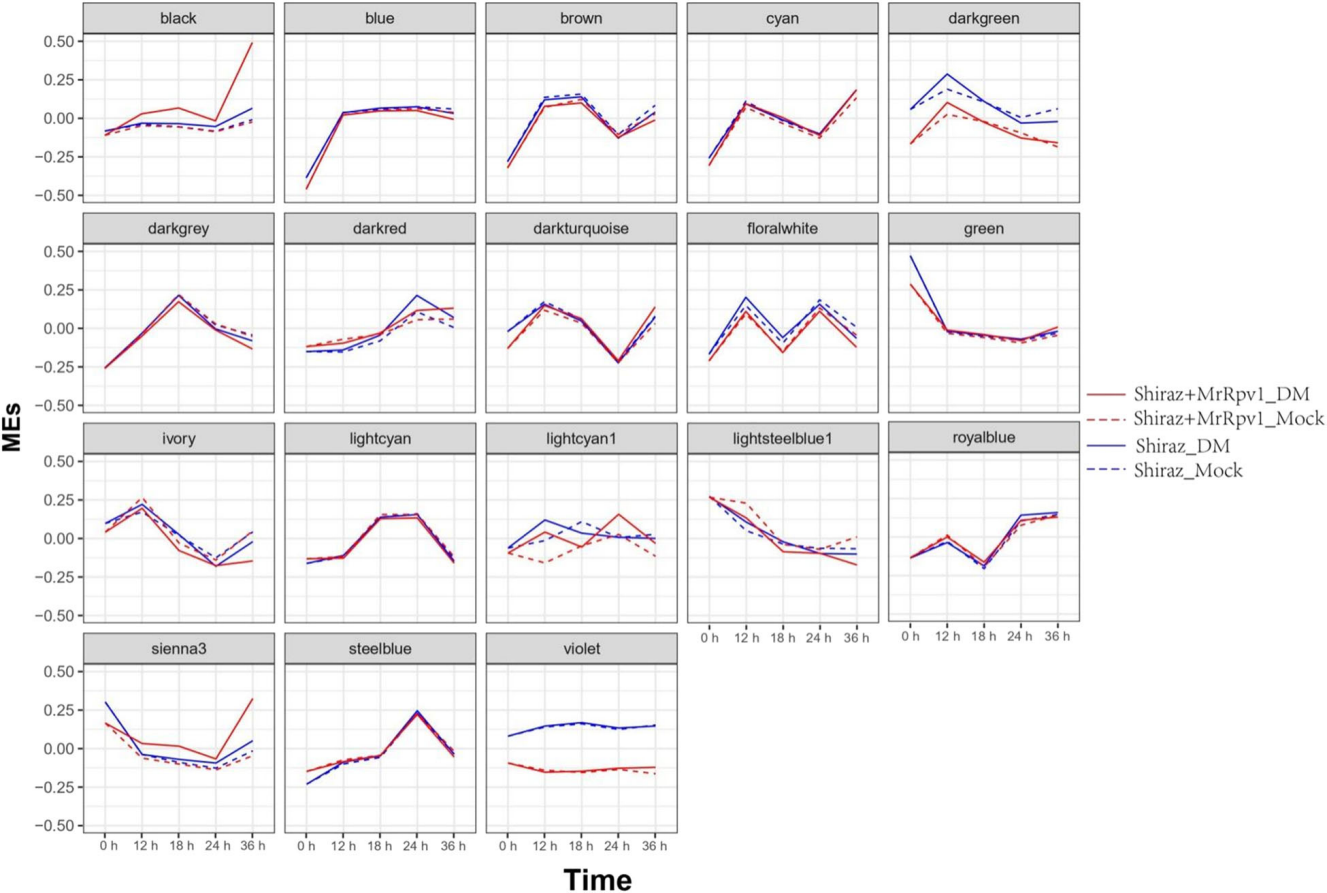
Expression Patterns of Coexpression Modules. Gene expression data in Shiraz and *MrRPV1*-transgenic Shiraz challenged with *Plasmopara viticola* and mock were subjected to WGCNA and grouped into modules containing genes with similar expression patterns. Expression levels of module eigengenes (MEs) (ordinate) that summarize gene expression levels in the modules are plotted over time (abscissa)

To gain insights into the regulation of gene networks by *MrRPV1* in response to DM infection, rather than focusing on individual genes, coexpression network analysis with WGCNA was performed on the basis of correlation patterns of the expressed genes across all 54 samples, i.e., S_DM, S_Mock, TS_DM, and TS_Mock, to find the hub genes related to *MrRPV1*-mediated resistance to *P. viticola* infection. Modules are defined as clusters of highly interconnected genes. This analysis resulted in 18 distinct modules (MEs) with different expression patterns (Fig. 3, Supplemental Data Set 9). Nine MEs, designated MEblack, MEblue, MEcyan, MEgreen, MEivory, MElightsteelblue1, MEsienna3, MEsteelblue, and MEviolet, showed statistically significant differences in expression in DM-infected *MrRPV1*-transgenic leaf tissues compared with mock-inoculated tissues at least one-time point. Notably, the MEblack and MEsienna3 modules were specific to the *MrRPV1*-transgenic line. A total of 1299 genes were clustered in these two modules, of which 65.7% (854/1299) were DEGs between *MrRPV1*-transgenic vs wild-type Shiraz lines. This means that most of the DEGs induced by the *MrRPV1* gene were concentrated in these two modules.

The expression patterns of the MEblack module genes in the mock treatment of leaf discs from *MrRPV1*-transgenic and wild-type Shiraz plants were relatively consistent across all time points. In wild-type Shiraz leaves, DM infection did not result in a significant change in the expression of MEblack module genes compared to that of the mock control. However, the expression of MEblack module genes increased within 12–18 hpi in the *MrRPV1-*transgenic plants, which was followed by an even larger increase in expression 24–36 hpi. The genes in the MEsienna3 module showed a gradual downregulation in expression for the first 24 hpi, followed by upregulation between 24 and 36 hpi, which was much more pronounced in Shiraz plants containing the *MrRPV1* gene. In the MEblack module, five out of the top 10 hub genes (Table 1) encode proteins thought to be involved in plant defense, including cysteine-rich receptor-like protein kinase 1 (VIT_17s0000g08720), pathogenesis-related protein PR-4 (VIT_14s0081g00030), two chitinases (VIT_16s0050g02210 and VIT_16s0050g02220), and a receptor-like protein kinase (VIT_01s0011g03990).

**Table 1:** Top 10 hub genes identified in the black module via WGCNA.

GeneID	KME	Annotation
VIT_17s0000g08720	0.989466	Cysteine-rich receptor-like protein kinase 1
VIT_08s0058g00860	0.986909	Protein LURP-one-related 15
VIT_14s0081g00030	0.977658	Pathogenesis-related protein PR-4
VIT_14s0083g00460	0.975961	Tryptophan synthase
VIT_00s0454g00010	0.973409	Subtilisin-like protease SBT1.7
VIT_16s0050g02210	0.972616	Acidic endochitinase
VIT_01s0011g03990	0.971902	Probable receptor-like protein kinase
VIT_17s0000g00940	0.971082	Putative F-box protein
VIT_15s0021g01270	0.970827	uncharacterized protein
VIT_16s0050g02220	0.970416	Acidic endochitinase

### Transcriptional regulatory modules associated with transcription factors

Transcription factors are critical in regulating transcriptional programs controlling plant development and responses when plants are confronted by phytopathogens^39^. The expression of a total of 77 transcription factors (TFs) assigned to 12 different families responded within the first 36 hpi with DM in *MrRPV1*-transgenic leaves (Supplemental Data Set 10). For instance, 20 ethylene-responsive transcription factors (*ERFs*) were specifically induced in *MrRPV1*-transgenic plants, 5 of which had a fold change >6.1. Notably, compared with that of the mock-inoculated *MrRPV1*-transgenic leaves, the expression of *WRKY11* (VIT_04s0069g00970) in *MrRPV1*-transgenic leaves increased dramatically, from 55.1-fold at 18 hpi to 401.1-fold at 36 hpi. In addition, the expression levels of three MYB TFs, *MYB15* (VIT_05s0049g01020), *MYB13* (VIT_05s0049g01010) and *MYB138* (VIT_12s0134g00490), were increased ~16.3-fold, ~39.8-fold and ~30.5-fold at 24 hpi and 36 hpi, respectively (Table S10).

**Fig. 4:**
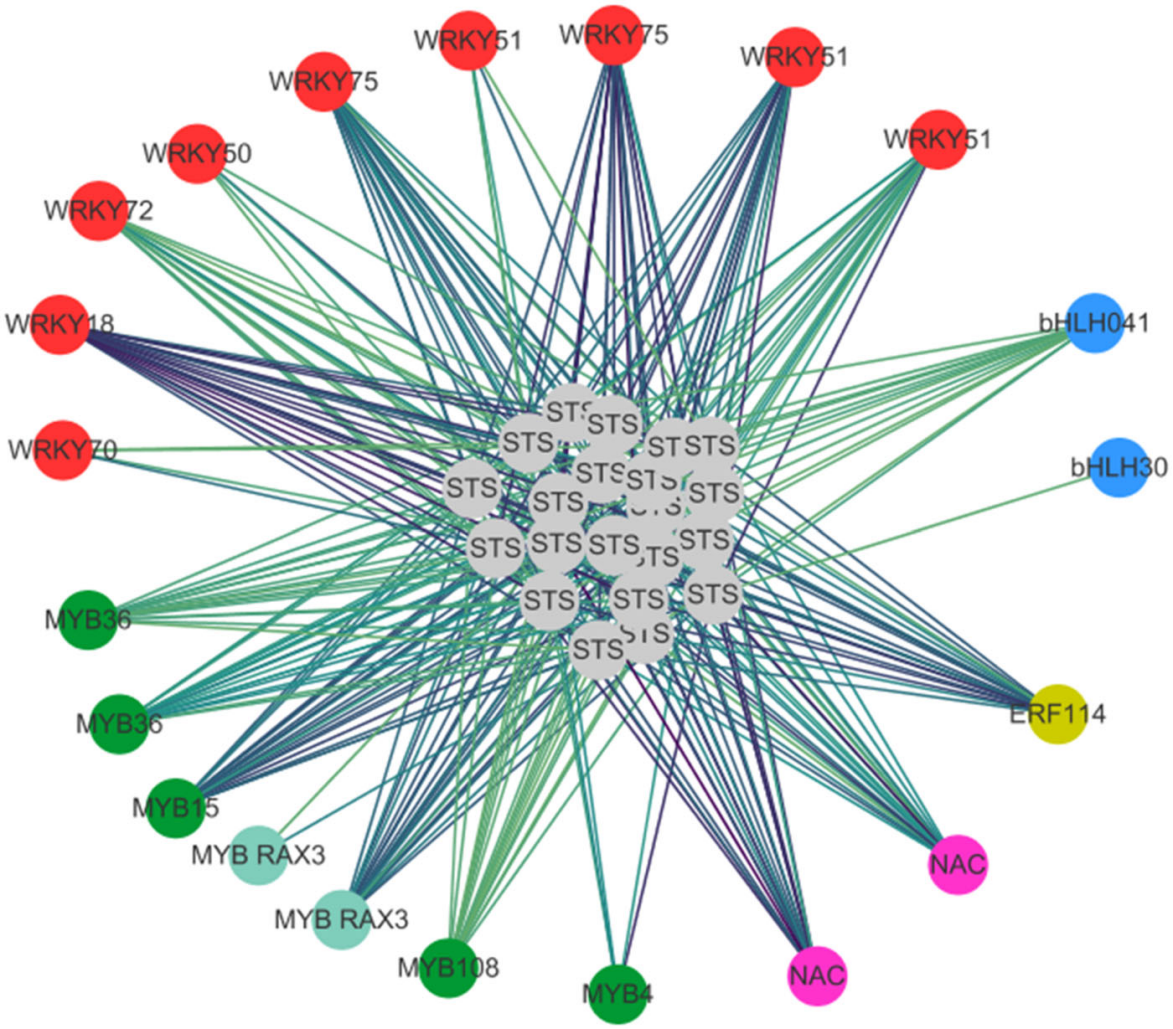
Gene coexpression network of grapevine stilbene synthase (*VvSTS*) and transcription factors (TFs) in black MEs. The edges signifying the correlations in the black module were filtered by the condition that the weight value was >0.2. Different TF families are represented by different colors

To investigate the regulatory network of TFs, we analyzed motif enrichment in the promoters of genes in each module compared with those of the genes in all the samples. We found that the different modules shown in Fig. 3 can be characterized by different sets of TF binding motifs. Interestingly, the MEblack cluster showed enrichment for genes with binding motifs of WRKY, MYB, and AHL transcription factors in their promoters, whereas MEsienna3 was enriched for genes with binding motifs for CMTA transcription factors. Furthermore, binding motifs for WRKY TFs were enriched only in the MEblack module, which is related to plant-pathogen interaction pathways. The WGCNA also identified a higher number of TFs, including WRKY, MYB, NAC, bHLH, and ERF, that were strongly coexpressed with *STSs* in the MEblack module (Fig. 4, Supplemental data set 11). All these findings suggest that transcription factors, especially WRKY TFs, play important roles in *MrRPV1*-mediated resistance.

**Fig. 5:**
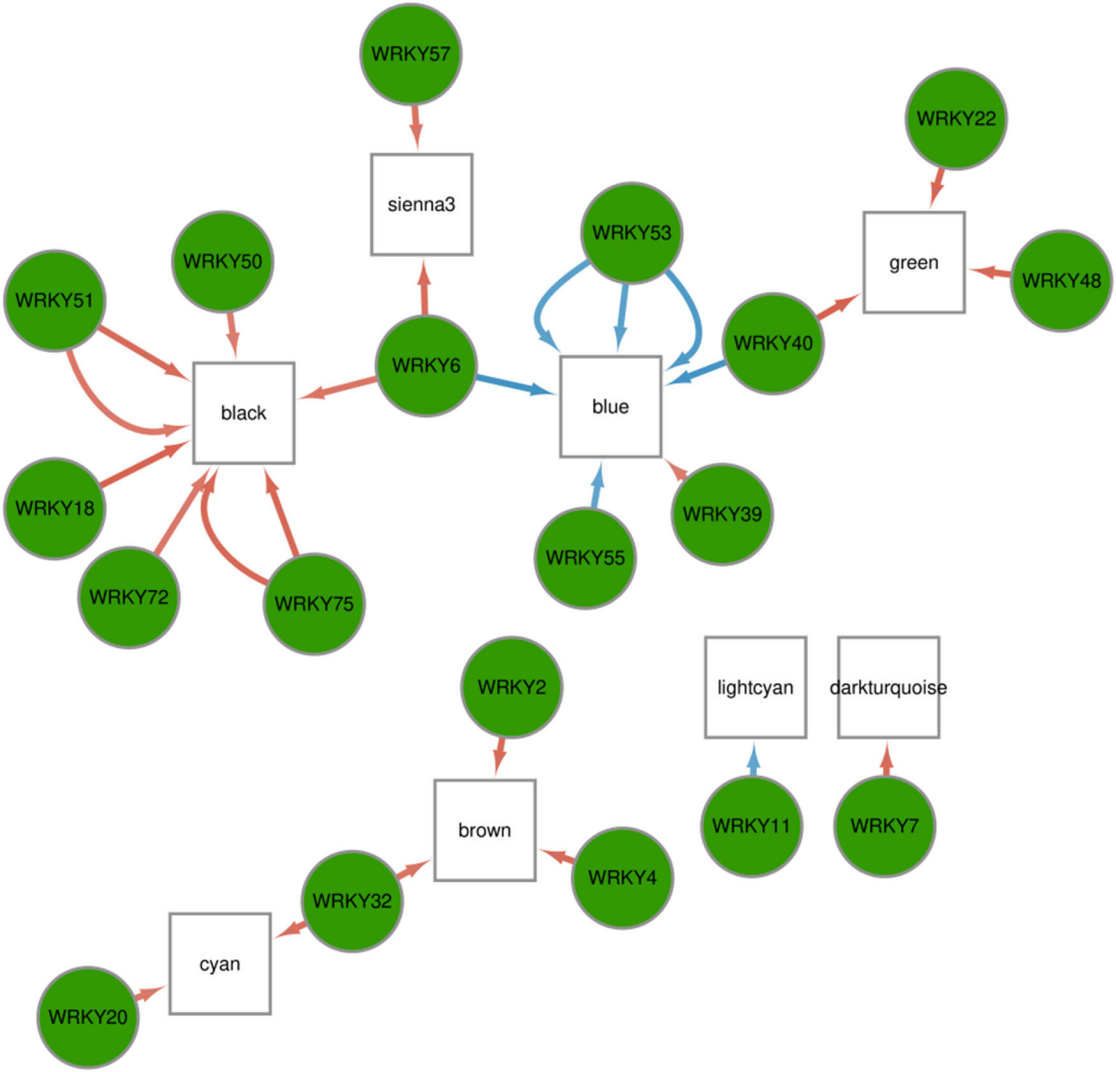
Coexpression relationships between MEs and WRKY transcription factors in wild-type Shiraz and *MrRPV1*-transgenic Shiraz challenged with *P. viticola*. White boxes indicate MEs, and green circles indicate transcription factors. Red arrows indicate positive correlations (kME > 0.8), and blue arrows indicate negative correlations (kME < −0.8)

To further investigate connections between these TFs and the *MrRPV1*-induced immunity network, the kME values, a measure of correlations between the expression patterns of individual genes and those of MEs, were calculated using WGCNA. We selected differentially expressed MEs and WRKY transcription factors to construct their coexpression networks (|kME| > 0.8) (Fig. 5, Supplemental data set 12). Six modules, including MEblack and MEsienna3, were positively correlated with multiple WRKY transcription factors, while MEblue was negatively correlated with most WRKY transcription factors. This suggests that the change in expression of multiple WRKY transcription factors contributes to upregulation or downregulation of the genes in these modules.

## Discussion

In this study, we utilized transgenic Shiraz plants carrying the *MrRPV1* resistance gene from *M. rotundifolia* to investigate the mechanism by which this gene confers resistance to DM. This approach allowed the precise identification of defense genes and pathways specifically linked to MrRPV1 activation because of the high level of uniformity of the genetic background of the transgenic and wild-type plants, except for the presence of this resistance gene. As expected, significantly fewer DEGs were identified using this approach than using comparisons of transcription profiles of DM-susceptible and DM-resistant grapevines with very different genetic backgrounds^20–22,25^. For example, we identified a total of 1356 DEGs that were differentially regulated in *MrRPV1*-transgenic Shiraz leaves in comparison to wild-type Shiraz leaves in response to DM infection. In contrast, a previous transcriptomic analysis that compared susceptible *V. vinifera* and resistant *V. riparia* plants following DM inoculation found as many as 5550 and 6379 genes with statistically significant differences in expression at 12 and 24 hpi, respectively^20^.

PCD is a fundamental cellular process in animals and plants. In plants, two forms of PCD, developmentally controlled PCD (dPCD) and pathogen-triggered PCD (pPCD), have been described^40^. The plant hypersensitive response (HR) is a rapid localized PCD that occurs at the point of pathogen penetration and is generally associated with ETI^41^. This PCD is an effective defense strategy against biotrophic plant pathogens by restricting pathogen access to water and nutrients. However, the HR is not restricted to the ETI response^42^. More recently, ETI was shown to restore and potentiate PTI signaling components, leading to a robust immune response, and PTI coactivation was shown to enhance NLR-mediated HR cell death^43,44^. In our study, the strongest resistance associated with the induction of PCD in penetrated cells was observed in Shiraz transgenic line S8-1, and this *MrRPV1*-mediated PCD induction was first observed 24 h post inoculation. The MrRPV1 protein recognizes the AvrRPV1 effector protein that is secreted by *P. viticola* and mediates the activation of the HR, leading to leaf resistance to DM. Downstream from NLR activation, the HR involves a series of events that include calcium influxes, accumulation of SA, ROS, and transcriptional reprogramming^45^. Many DEGs related to most of these elements were identified in *MrRPV1*-transgenic Shiraz. Because most of these elements are shared between PRR and NLR signaling, the zigzag model of the plant immune system defined a threshold for the activation of HR^2^ rather than support its being a highly regulated phenomenon. However, recent advances proposed a provocative model in which the funnel-like structure of a resistosome triggers HR cell death by translocating into the plasma membrane and perturbing membrane integrity, similar to the action of pore-forming toxins^46^.

Plant hormones play central roles in the process of plant resistance to many pathogens. It is generally accepted that SA and JA/ET induce defense against biotrophic and necrotrophic pathogen attack, respectively^47^. However, studies in the past few years have proven that JA/ET-mediated defenses also contribute to resistance against some biotrophic pathogens^48,49^, although the signaling crosstalk between them is not clear. In the present study, the induction of LOX, AOS, ACO, ACS, and ERF genes involved in JA/ET signaling and biosynthesis was quantitatively greater in degree in *MrRPV1*-transgenic plants than in wild-type Shiraz plants from 18 hpi to 36 hpi. The results of other studies have also previously implicated a role for JA in the genetic and inducible resistance of various grapevine species and varieties to DM^20,48–55^. *PR1* gene expression is associated with the induction of disease resistance in plants. It is a molecular marker for disease resistance mediated by salicylic acid^56^. In our study, the expression of *PR-1* genes increased up to 480-fold, suggesting that *PR-1* genes may also play an important role in *MrRPV1*-mediated resistance. In Arabidopsis, the EDS1-PAD4 complex predominantly contributes to TNL-based ETI^57^. Recent studies have shown that TNL-mediated resistance responses require EDS1 complexes incorporating a SAG101 isoform in Solanaceous species but not in members of the Brassicaceae^58,59^. Intriguingly, our results showed that the *SAG101* genes were differentially expressed 18 h earlier than the *PAD4* gene. Thus, we propose that SAG101 may be required for *MrRPV1*-mediated immune signaling in grapevine. In *Arabidopsis thaliana*, the combined action of EDS1, PAD4, and SAG101 can also promote SA-mediated defenses to limit *Fusarium graminearum* infection^60^. Altogether, we speculate that multiple phytohormones participate in the *MrRPV1*-mediated defense against DM infection.

A variety of secondary metabolites play a vital role in the host plant’s immune process against pathogens, including terpenoids, alkaloids, flavonoids, and phenolics^38,61^. Previous studies have shown that stilbenes confer resistance to grape powdery mildew by efficient recruitment of SA signaling^62–64^. Multiple omics data revealed that *Rpv3-1*-mediated resistance to grapevine DM is associated with accumulation of stilbenes^23,24^. Genome-wide analysis of the grapevine stilbene synthase gene family identified three principal groups designated *STS* A, *STS* B, and *STS* C^65^. In our study, the expression of genes in groups *STS* A and *STS* C showed a continuous increase within 36 h after DM inoculation in *MrRPV1*-transgenic leaf discs. In addition, upregulation of *PAL* and *ROMT* genes involved in the stilbenoid biosynthesis pathway was also observed in resistant plants. Our results strongly suggest an important role for stilbenes in *MrRPV1*-mediated defense against DM, as has been proposed previously for the DM-resistance loci *Rpv3*^23^ and *Rpv10*^15,22^. The results of our gene network analysis are also consistent with the results of previous studies showing that WRKY and MYB transcription factors strongly coexpress with *STS* genes in grapevine tissues under biotic and abiotic stress^66–68^.

## Materials and methods

### Plant and pathogen materials

Stably transformed transgenic Shiraz plants containing *MrRPV1* were generated previously as described by Feechan et al.^18^. Untransformed and transgenic *V. vinifera* cv. Shiraz (clone BVRC12) plants were grown in a greenhouse (16 h light, 26 °C/8 h dark, 22 °C). *Plasmopara viticola* inoculum was collected from an experimental vineyard on the Waite Campus, Adelaide, Australia, and then maintained on detached leaves of *V. vinifera* cv. Cabernet Sauvignon. Discs 1–1.5 cm in diameter were cut from fully expanded leaves (collected from nodes 3–5), sprayed with a *P. viticola* sporangia suspension in water (5 × 10^4^ sporangia/ml) and incubated in sealed petri dishes in a chamber at 22 °C under a 16-h light/8-h dark cycle for 5–6 days.

### RNA-seq and data analysis

A total of six clonal copies of untransformed Shiraz and six clonal copies of *MrRPV1*-transgenic Shiraz line S8-1^18^ were divided into three groups of two “duplicates” each. Two leaves from each duplicate plant were selected and individually bagged, producing three control groups and three transgenic groups. From each group of leaves, a total of 60 discs were cut and placed onto moist filter paper with the abaxial side facing up. Prior to inoculation, six discs from each of the six groups (three controls, three transgenic) were selected at random from the 60 and snap frozen in liquid nitrogen to act as zero-time controls. Within each group, half of the discs were sprayed with water, and the other half were sprayed with a *P. viticola* suspension. Six discs were harvested at random from the mock and *P. viticola*-inoculated treatments from the three control and three transgenic groups at 12, 18, 24, 36 hpi, snap frozen in liquid nitrogen and stored at −80 °C until use. Total RNA was isolated with the Spectrum Plant Total RNA purification kit (Sigma Aldrich) following the manufacturer’s instructions.

A total of 3 μg of total RNA per sample was used as input material for RNA sample preparations. Sequencing libraries were generated using the NEBNext® Ultra™ RNA Library Prep Kit for Illumina® (NEB, USA) following the manufacturer’s recommendations, and index codes were added to attribute sequences to each sample. Library construction and RNA sequencing were performed by the Novogene Bioinformatics Institute (Beijing, China). Indexing of the reference genome was undertaken using Bowtie v2.0.6, and paired-end clean reads were aligned to the reference Grapevine genome PN40024 (GenBank assembly accession No: GCA_000003745.2) using TopHat v2.0.9. HTSeq v0.6.1 was used to count the read numbers mapped to each gene. Differential expression analysis of two conditions/groups (two biological replicates per condition) was performed using the DESeq R package (1.10.1). The resulting *P*-values were adjusted using Benjamini and Hochberg’s approach^69^ to control the false discovery rate. Genes with an adjusted *P*-value < 0.05 were assigned as differentially expressed. GO enrichment was performed using the GOseq R package^70^. KOBAS software was used to test the statistical enrichment of differentially expressed genes in KEGG pathways^71^.

### WGCNA

Coexpression networks were constructed using the R package WGCNA^72^. Gene expression values of all samples were used to construct a single hybrid network. After excluding genes with FPKM > 1 across 50% of the samples, expression values of 19,732 genes were imported into WGCNA to construct coexpression modules using the automatic network construction function blockwiseModules with default settings, except that the power was 6. Genes were clustered into 18 correlated modules (correlation >0.8). The networks were visualized using Cytoscape^73^. For motif enrichment analysis using AME^74^, the 1000 nt upstream of the transcription start sites of the members of 18 modules were tested for enrichment of known cis-elements with the set of genes from all the modules as the control. The eigengene-based gene connectivity, kME, was calculated using the signedKME function in the WGCNA package and was used to visualize relationships between transcription factors and expression patterns of module eigengenes using Cytoscape. Genes with high positive kME values were referred to as intramodular hub genes and centrally located in their respective modules.

### Gene expression analysis

Total RNA isolated from leaves of the untransformed and *MrRPV1*-transgenic Shiraz plants, mock-inoculated or inoculated with *P. viticola*, was used for cDNA synthesis using HiScript® II Q RT SuperMix for qPCR (+gDNA wiper) (Vazyme Biotech Co. Ltd. Nanjing, China). Real-time PCR was performed using a LightCycler® 480 II Real-time PCR Instrument (Roche, Switzerland) with a 10 μl PCR mixture that included 1 μl of cDNA, 5 μl of 2× QuantiFast® SYBR® Green PCR Master Mix (Qiagen, Germany), 0.2 μl of forward primer, 0.2 μl of reverse primer and 3.6 μl of nuclease-free water. Reactions were incubated in a 384-well optical plate (Roche, Switzerland) at 95 °C for 5 min, followed by 40 cycles of 95 °C for 10 s and 60 °C for 30 s. Each sample was run in triplicate. Specific primers for the selected genes are listed in Supplemental Table 1. The expression levels of mRNAs were normalized to that of EF1α and were calculated using the 2^−ΔΔCt^ method^75^.

### Accession numbers

Sequence data from this article can be found at the NCBI repository under the following accession number: PRJNA706058.

## Supplementary information

Venn diagram of differentially expressed genes in S_DM vs. S_Mock and TS_DM vs. TS_Mock

Relative expression levels of DEGs from real time qRT-PCR compared with RNAseq

Supplemental Table 1

Dataset 1

Dataset 2

Dataset 3

Dataset 4

Dataset 5

Dataset 6

Dataset 7

Dataset 8

Dataset 9

Dataset 10

Dataset 11

Dataset 12
