# Supplementary figures and images for: Transcriptional profiling reveals multiple defense responses in downy mildew-resistant transgenic grapevine expressing a TIR-NBS-LRR gene located at the MrRUN1/MrRPV1 locus
Source: Hortic Res. 2021 Jul 1;8:161. doi: 10.1038/s41438-021-00597-w (PMC8245497; doi:10.1038/s41438-021-00597-w)

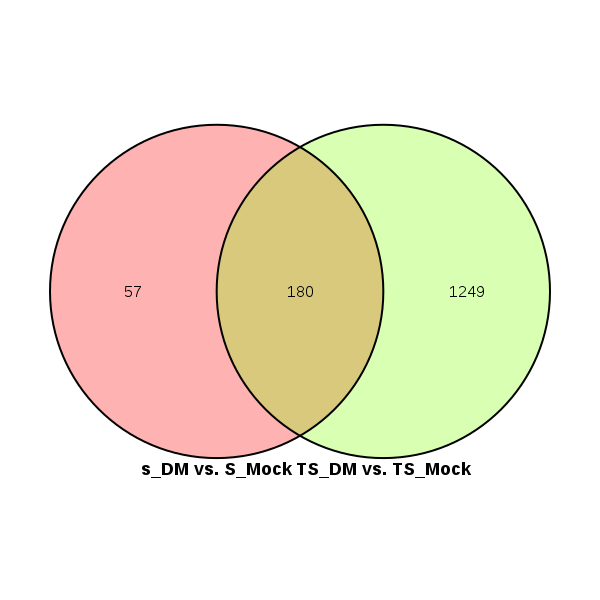

Supplement: Supplementary file 1 — Venn diagram of differentially expressed genes in S_DM vs. S_Mock and TS_DM vs. TS_Mock [file 41438_2021_597_MOESM1_ESM.png]

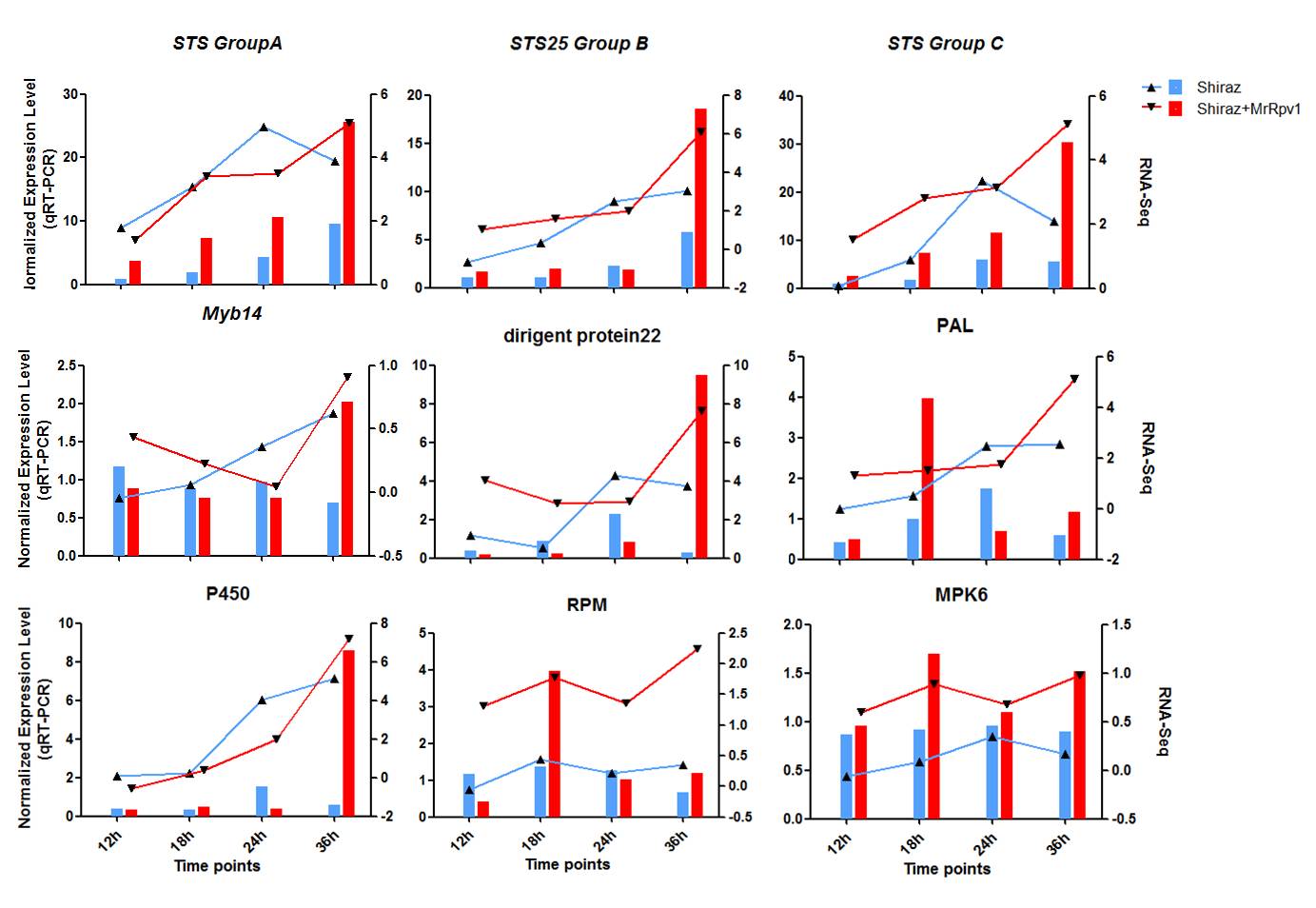

Supplement: Supplementary file 2 — Relative expression levels of DEGs from real time qRT-PCR compared with RNAseq [file 41438_2021_597_MOESM2_ESM.jpg]
